# Mobility Management of Unmanned Aerial Vehicles in Ultra–Dense Heterogeneous Networks

**DOI:** 10.3390/s22166013

**Published:** 2022-08-12

**Authors:** W. T. Alshaibani, Ibraheem Shayea, Ramazan Caglar, Jafri Din, Yousef Ibrahim Daradkeh

**Affiliations:** 1 Electrical Engineering Department, Faculty of Electrical and Electronics Engineering, Istanbul Technical University (ITU), 34469 Istanbul, Turkey; 2 Electronics and Communication Engineering Department, Faculty of Electrical and Electronics Engineering, Istanbul Technical University (ITU), 34469 Istanbul, Turkey; 3 Wireless Communication Centre, School of Electrical Engineering, Faculty of Engineering, Universiti Teknologi Malaysia, Johor Bahru 81310, Malaysia; 4 Department of Computer Engineering and Networks, College of Engineering in Wadi Alddawasir, Prince Sattam bin Abdulaziz University, Al-Kharj 11991, Saudi Arabia or ibr.shayea@gmail.com (I.S.)

**Keywords:** connected drones, 5G networks, UAV, machine learning, handover, mobility management, deep learning, drones, heterogeneous 6G networks

## Abstract

The rapid growth of mobile data traffic will lead to the deployment of Ultra–Dense Networks (UDN) in the near future. Various networks must overlap to meet the massive demands of mobile data traffic, causing an increase in the number of handover scenarios. This will subsequently affect the connectivity, stability, and reliability of communication between mobile and serving networks. The inclusion of Unmanned Aerial Vehicles (UAVs)—based networks will create more complex challenges due to different mobility characterizations. For example, UAVs move in three–dimensions (3D), with dominant of line–of–sight communication links and faster mobility speed scenarios. Assuring steady, stable, and reliable communication during UAVs mobility will be a major problem in future mobile networks. Therefore, this study provides an overview on mobility (handover) management for connected UAVs in future mobile networks, including 5G, 6G, and satellite networks. It provides a brief overview on the most recent solutions that have focused on addressing mobility management problems for UAVs. At the same time, this paper extracts, highlights, and discusses the mobility management difficulties and future research directions for UAVs and UAV mobility. This study serves as a part of the foundation for upcoming research related to mobility management for UAVs since it reviews the relevant knowledge, defines existing problems, and presents the latest research outcomes. It further clarifies handover management of UAVs and highlights the concerns that must be solved in future networks.

## 1. Introduction

The rapid growth of wireless technology has caused a dramatic shift in people’s daily lives. Mobile–connected devices, connected applications, Machine to Machine (M2M), Internet of Things (IoT), and other services are steadily increasing. IoT connects almost everything throughout numerous environments. With its evolution, it will be the most utilized technology and the largest telecom market. IoT marks a new era of total automation and offers efficient solutions for several fields. Since it has become extremely simple to connect several devices in different locations, its impact on daily life has been tremendous. Various industries are currently demanding wide–area communication, especially for numerous operations that are performed indoors [1,2,3,4,5]. These factors will further lead to the massive growth of mobile data traffic.

The transmission and reception of signals by antenna systems are critical components of wireless technology (such as IoT, autonomous aerial vehicles, and wireless communication systems). Antenna systems are used for transmitting and receiving signals in wireless communication systems. Tiny miniature antennas are now in high demand for various applications, including communication systems, radio sensors, etc. Several studies have been conducted in this field. For instance, in ref. [6], a strategy was suggested for reducing the size of planar antennas by integrating loaded cell (LC) elements. The planar transmission model can then be applied to analyze two different antenna configurations. This model enables preliminary design research and enhances the comprehension of parametric structural relationships. In ref. [7], a new printed leaky–wave antenna (LWA) was also designed, produced, and characterized with beam routing. The antenna design procedure was based on the placement of an appropriate number of E–shaped arms on printed circuit boards, resulting in an operating bandwidth of 118.7 GHz. This topic generated a wide range of valuable research results. Although this paper cannot discuss all outcomes, a link is provided for researchers to access the most significant studies in this field [8,9,10,11,12,13,14,15].

In recent years, the urgent need to apply large frequency bands to enable quick and smooth data transfers has emerged, especially in light of the advanced technology provided by the fifth generation (5G) and sixth generation (6G) networks. Establishing airborne communication will mark the advanced stage of development for communication networks. The number of obstacles and scattered objects impeding data transmission will significantly reduce. This network type is typically employed in emergency scenarios, for instance, when communication infrastructure is unavailable, during natural disasters, or in areas where conventional communication networks are too expensive [16,17]. Other high mobility–based emergency services are also required, such as for provision of medical assistance to patients in ambulances struggling with life–threatening situations before they reach a hospital where competent medical care is available. Real–time consultations with specialists in remote hospitals should also be made possible. These services are urgently needed, especially in the current pandemic scenario [18,19].

New telecommunications seek faster data rates, lower latency, higher quality benefits, and increased user capacity [20]. When viewing cellular service maps, cellular coverage is unavailable in more than 60% of locations to several reasons. Firstly, it would be inefficient to deploy fixed base stations (BSs) in remote areas where there is limited human activity, and, although greater human activity requires remote management, complex terrains may hinder BS deployment. Stationary BSs may struggle to handle excess information traffic, especially when distant requests arrive in an unanticipated or unpredictable manner [21,22]. Future telecommunication generations and beyond must establish additional coverage alternatives to provide on–demand and remote services for increasing gadget use. Cellular networks supplemented with UAVs are referred to as UAV–supported cellular systems [23]. The next generation of UAV–BS have generated considerable interest due to their rapid deployment, mobility, extensive opportunities for unobstructed propagation channel, and resilient features. UAVs will therefore play a significant role in future mobile communication networks, serving as BSs and mobile users in the sky. UAVs can be categorized into two classes: those that operate autonomously, and those that supplement or assist overcrowded BSs [24,25,26,27]. Determining the optimum technology for UAV deployment is a critical and challenging issue that must be addressed. To resolve this issue, the first step is to provide on–demand services to geographically dispersed UEs. Given the probability of disaster and the necessity for UEs to have end–to–end communication, a powerful UAV spine network is required. Secondly, UAVs must also maintain connectivity to established BSs for backhaul connections and global data interchange. Overcoming these obstacles must be the first priority, particularly in the area of mobility management.

Several studies have been conducted on the use of UAVs as flying BSs. Various objectives were discussed, such as reducing the number of UAVs at different user densities to provide maximum coverage with the least amount of transmission control [28,29,30,31]. These studies overlooked the network and/or strength of the UAV spine organization, which is crucial in a complex environment. UAVs are regularly dispatched near established BSs to increase capability and enhance user satisfaction [32,33,34]. Most studies did not consider the links between UAVs and fixed BSs, which are crucial for providing backhaul connections. In conclusion, previous research [28,29,30,31,32,33,34] did not thoroughly examine the issue of UAV organization in cellular networks.

UAVs are aircraft that can autonomously fly without human guidance. This type of aircraft employs radio waves to navigate and present a route map. UAVs range in size, weight, shape, and engine. They are employed for specific purposes such as surveillance, gaming, spying, warfare, and presentations. As a result, they are furnished with technical gadgets such as cameras and Global Positioning System (GPS) sensors, both of which are necessary for monitoring and tracking. UAVs have a significant advantage in this area since they can immediately register and monitor any region or item without requiring additional infrastructure.

Based on the 3rd Generation Partnership Project (3GPP) TS 22–261, governments and corporate sectors are expected to use UAVs in a wide range of applications. The key issues of the future 6G network will be latency and dependability. UAVs will require more precise position information as well as protection against theft and fraud. The information transferred between UAVs and their control units must be secure. The next–generation mobile network must also be resistant to spoofing and non–repudiation to fully integrate UAVs. Unmanned Aerial System Traffic Management (UTM) is a centralized system for identifying, tracking, and authorizing UAVs and controllers. The UTM stores all identifications and metadata for UAVs and UAV controllers. The data interchange protocols used by UTM and mobile network centers, particularly Allied Telesis Management Framework (AMF), have permitted the confirmation and authorization of UAVs within the zone. Including UAVs in this flexible network will increase the AMF’s computational load. The use of UAV–mounted BS (UxNB) to extend the scope range is specified in 3GPP references. The UxNB may connect to a 5G core as a BS on the ground via a wireless backhaul link. The UxNB can be used in various situations (such as in emergencies, the temporary scope for UEs, and hotspot events) due to its quick setup and vast range of capabilities. When acting as a BS, the UxNBs must be validated by the center setup. Since UAVs have limited power, one condition for utilizing a BS is to consume as little energy as possible. The use of UAVs is limited due to their flying time and energy requirements. In conveyance administrations, for instance, using a single UAV results in a waiting period for the vehicle to return to base. As a result, UAVs should be used in swarm mode. Group management is the most basic requirement for a swarm of UAVs. Group management entails collecting confirmation and guaranteeing secure communication within a group.

This research focuses on the HO of UAV communication through wireless communication. A smooth HO is difficult to achieve while using traditional wireless networks. When compared to cellular networks, UAV wireless communications have less communication coverage and a longer HO procedure. The conventional HO technique further assumes that the coverage area for different cells is the same, which is not the case with UAVs due to their varying heights. The HO of UAVs should be more closely and efficiently monitored than that of terrestrial UEs. The use of traditional HO methods and strategies may not be suitable for UAVs. Although numerous relevant arrangements have been discussed throughout the literature, the problem remains unaddressed. Since future mobile networks are expected to be self–sufficient, node mobility forecasting may be a critical technique for optimizing the benefits of UAV systems. A large number of contemporary arrangements follow distance–based assumptions [2,35].

The objective of this study is to highlight the mobility management of connected UAVs in future mobile networks (5G and 6G). The article covers current research efforts devoted to addressing the inherent difficulties of using UAVs. The main research goal is to answer the most important questions in wireless communications. For example, why is HO difficult for UAVs, even more so during UAV mode when they can move freely in 3D? What are the current solutions to this problem? What are the future research directions in this field? This paper includes an assessment of the most significant practical solutions for resolving these problems. The central issues are outlined, and the recent research is highlighted and discussed. This paper extensively reviews the necessity of integrating UAVs into modern wireless communication networks, providing scholars with abundant knowledge in this field.

The remainder of this work is organized as follows. Section 2 provides an overview of the relevant literature. Section 3 highlights important achievements in the field and presents background research information. Section 4 focuses on the research challenges. Section 5 reviews the published works related to this research. Section 6 provides the proposed solutions. Section 7 discusses future research directions. Finally, Section 8 concludes this paper.

## 2. UAV Technology in Wireless Communication

Connected UAVs will be a revolutionary invention that will provide a wide range of services throughout various settings. The requirement for constant connection while on the move is a key issue that must be addressed. Defining the concept of UAVs and HO management is essential. This section provides an overview of UAVs, UAV communication networks, the HO concept, and 3D parameters. The following subsections present an extensive summary of the various subtopics.

### 2.1. Overview of UAVs

The use of UAVs has skyrocketed in recent years and continues to do so across multiple industries and services. UAVs present low–cost solutions in several industries, such as healthcare and marketing. They can provide a wide range of solutions for different scenarios. At this stage, it is crucial to employ cutting–edge technology to ensure the safe functioning and administration of this developing innovation. For decades, billions of devices have been linked together on the ground. Now, they are ready to be linked in the sky. Currently, UAVs can serve as wireless communication BSs to connect mobile users. However, several challenges will arise with connected UAVs before achieving reduced latency, enhanced connection dependability, real–time data transfer, and remote installations. The widespread adoption of contemporary developments, such as IoT and machine–to–machine communication (MTC), has significantly increased the number of UEs and MTC devices that interfere with mobile systems. As the number of UEs inside a BS scope increases, the quality of service (QoS) decreases. UxNB can be a viable solution in regions with a high concentration of UEs, such as stadiums. UxNB is a promising technology that can be applied in future for capacity injection due to its fast transmission. However, this new technology also possesses several security risks. When using UxNB for capacity injection, common verification, the development of a communication link between terrestrial BS and UxNB, and quick HO procedures may all raise security problems. This new protocol also suggests that the UE transition from earthbound to UxNB should be accomplished in groups.

UAV operations are primarily conducted at low altitudes in uncontrolled airspace. This airspace, which is regularly used for a range of existing flying exercises, contains critical infrastructure and is susceptible to changing conditions. In 5BG, the AMF, the radio access network (RAN), and the UE are the most important components. The AMF is in charge of registration, managing connections, ensuring that UEs can be reached, and managing their mobility. With 5G networks, the speed can reach up to 500 km/h, and with 6G networks, it will be even faster. This network function makes it possible to handle the mobility of nodes. Radio transceivers are used by RAN to connect to cellular networks. The BSs connect the UE to the New Radio (NR) user plane and control plane protocols.

3GPP defines UE as a device used by an end user to communicate with another user or service.

**Figure 1:**
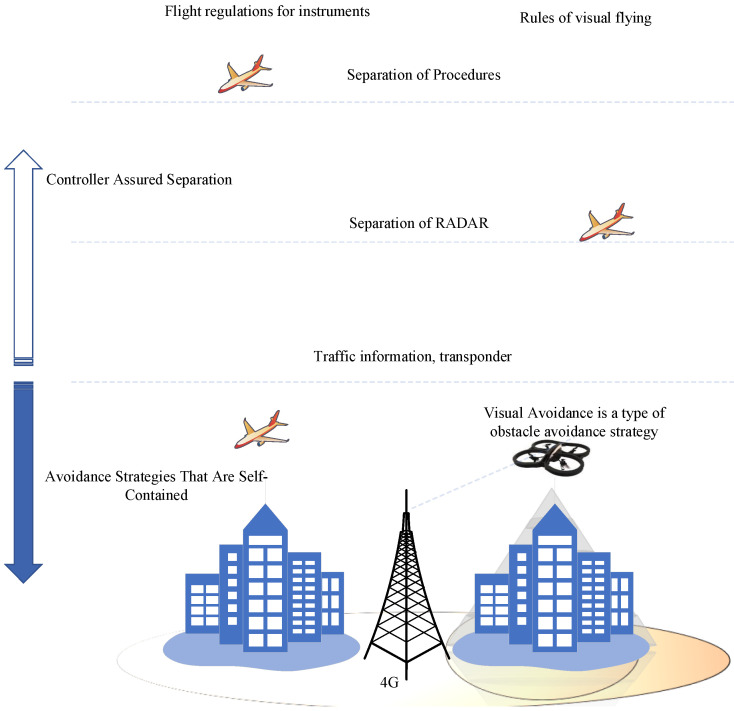
Details of the task confronting administrative bodies and the activity required for a comprehensive UTM framework.

Most pilots employ Visual Flight Rules (VFR) when flying in low–altitude airspace, as shown in Figure 1. Under VFR, each pilot is responsible for avoiding other aircraft or obstructions by maintaining a steady view of the region and other airspace users. Significant dangers associated with UAV movements are present in unclassified airspace if airframes are not monitored and human pilots are not present. The risk of bird collisions, building collisions, or accidents with other unmanned vehicles can cause significant issues among national aviation authorities. Collision avoidance frameworks will enhance the safety of unmanned aircraft. However, they are not designed to handle complex activities or movements of other planes and objects within the area. A new perspective is required to organize and monitor activities in low–altitude and unclassified airspaces. Several researchers are currently examining various methods to tackle the UTM challenge. Figure 1 presents the problem that administrative authorities must confront as well as the tasks required for a complete UTM framework. UTM is a traffic management ecosystem for movements that are not monitored by the Federal Aviation Administration’s (FAA) Air Traffic Management (ATM) system. The UTM will be improved and developed to define the services and responsibilities assigned to UAV operations when flying at low altitudes without supervision. Information exchange protocols and other technical details will also be specified in control and communication operations.

UTM is the mechanism that manages airspace to facilitate and permit UAV operations performed outside the beyond visual line of sight (BVLoS) where standard air services are unavailable. As a result, UAV operators and the FAA will work together to determine and report the state of the airspace in real–time. The FAA now imposes several restrictions on UAV operators to ensure safe management operations. FAA and UAV operators mostly communicate through a distributed network of highly automated systems via the Application Programming Interfaces (APIs). They do not coordinate through verbal communication, as pilots and air traffic controllers do.

### 2.2. UAV Communication Network

The IEEE 802.11 Wireless Local Area Network (WLAN) and radio technology both conduct command and control activities for most commercial UAVs. However, due to the UAV’s speed and fluctuating altitudes, IEEE 802.11 is unable to meet the required conditions. Command and control activities can be accomplished in a non–licensed range; however, numerous security and reliability issues would arise. Cellular networks are the only option. Cellular networks are stable, secure, and capable of covering wide areas with acceptable data speeds. However, they are not designed to support flying devices despite substantial standardization efforts. The most pressing issues continue to be interference and radio coverage. Certain limits must be met when a cellular network is linked with a UAV to improve coverage and capacity. UAVs are used as relays or mobile BSs to enhance coverage, connectivity, and capacity. RANs are also simple to install in regions where no established network architecture is available. This implementation is a configuration style that can be set up in the event of a disaster to avoid investing time and money on new infrastructure. It is also beneficial for increasing capacity and coverage during significantly crowded gatherings, such as concerts and sporting events [2,36,37,38].

### 2.3. Antenna Tilting and Cell Association

**Figure 2:**
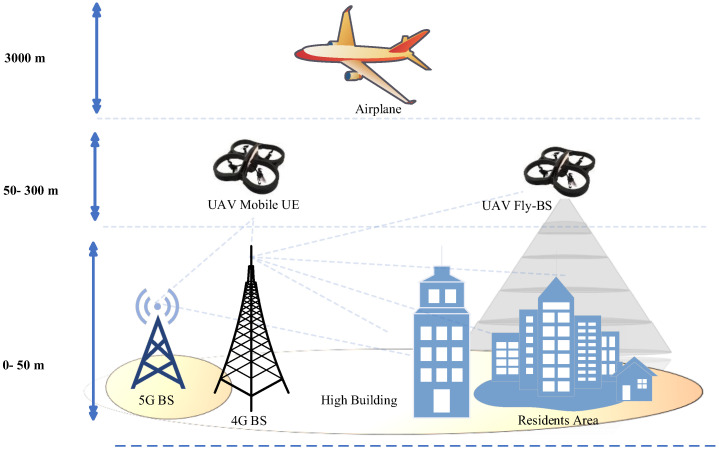
UAV base station (UBS) and normal BSs in a future ultra–dens heterogenous network.

**Figure 3:**
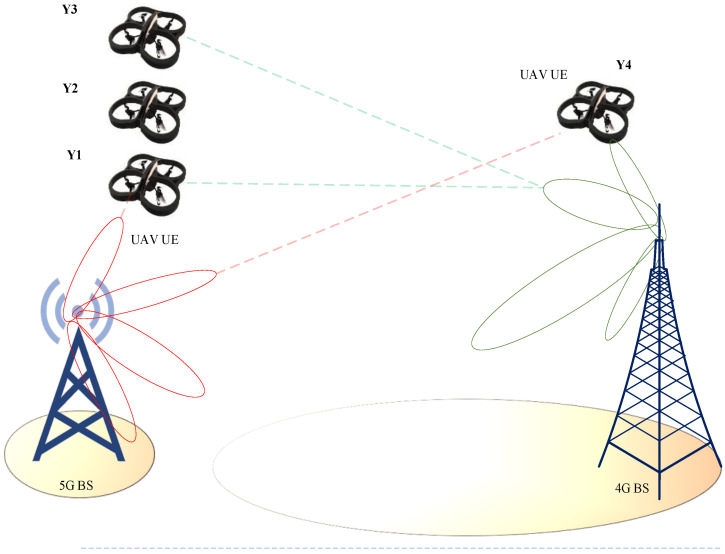
UAV connection via side lobes in a future ultra–dens heterogenous network.

To provide the best service to ground users, cellular BS antennas are tilted downwards. Aerial coverage has recently received significant attention, mostly for connecting airline passengers on domestic flights. Only a small number of BSs with upgraded antennas are necessary to ensure extensive coverage and continuous connectivity during the flight. However, due to construction and regulatory constraints, these methods cannot be used for commercial UAVs which frequently fly at lower altitudes, such as 50–300 m, as illustrated in Figure 2. UAVs are fundamentally different from terrestrial users since the assumptions that apply to terrestrial users are not applicable to aerial users. Consider the following example, two BSs (A and B) have antennas tilted downwards with the primary lobes facing down towards the earth. The ground user connects to the BS. If the signal strength from both BSs is equal, the user will stay connected to the previous one. In the case of UAVs, side–lobe antennas are useful. Figure 3 shows that despite being closer to BS A than BS B, the UAV at Y1 is served by BS B. This will cause excessive HOs and ping–pong effects. This issue also applies to horizontal locations.

For locations Y2 and Y4 in Figure 3, the picture can be expanded to include a large number of BS s, signifying that the rate of HO for UAVs will be more excessive than conventional or traditional networks. Increasing the UAV’s height will decrease the competitiveness of its service via the main lobes as long as the terrestrial BS antennas are slanted downwards. As a result, the service given to UAVs at high altitudes will be via the side lobes, which is not at the same level offered by the main lobes. Due to the increased potential of line–of–sight (LoS) at such high altitudes, UAV communication will suffer from uplink (UL) and downlink interference. This will create severe interference and navigation management issues. Increasing the altitude will allow the side lobes of the BS antennas to have more than one connection possibility depending on the location of the UAV. This raises the possibility of LoS communication links, which increases interference in neighboring cells when compared to UE ground equipment [39,40,41,42,43].

### 2.4. UAV Communication Scenarios

From a wireless perspective, a UAV in a 3D environment could potentially act as a mobile BS and mobile EU. Detailed consideration of both of these scenarios is provided below.

#### 2.4.1. Flying Base Stations

A flying BS that connects backhaul and access networks can be a UxNB. The so–called fly ad–hoc network (FANET) is formed when more than one UAV is included in a transmitting apparatus. FANETs are air–borne frames for remote wireless ad hoc networks (WANETs) or mobile ad hoc networks (MANETs). An innovative aspect of the 5G network is “network from the sky”. UAV have the ability to provide on–demand systems to specific regions due to their built–in mobility features, flexibility in three–dimensional space, adaptive elevation, and symmetric revolution. Ground users can benefit from premium services such as high–quality wireless connections, seamless connection, large data capacity, and low degradations thanks to these unique characteristics. UAV integration with distant cellular systems serving as aerial communication platforms will open up previously unconsidered foundations, new perspectives, and numerous possibilities [44].

**Figure 4:**
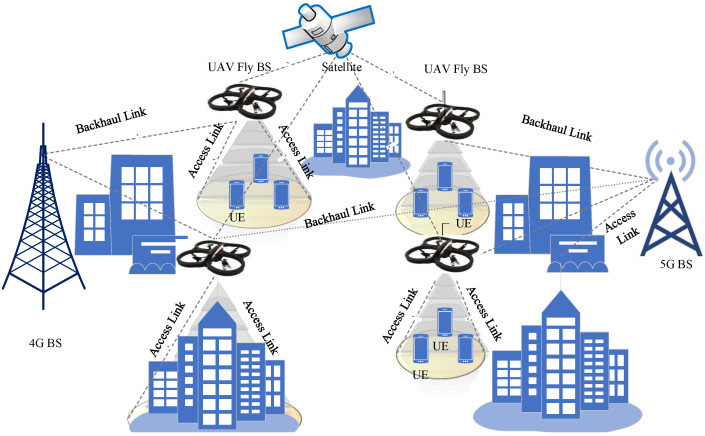
UAVs as base stations in a future ultra–dens heterogenous network.

When compared to their earthly counterparts, several differences are unquestionably present. The average height of earthbound BSs in an urban setting is about 10–20 m. UAVs can hover up to 100–120 m. This allows the UAV to have a longer range than traditional terrestrial BS s, further reducing interference from nearby terminals. Ground terminals are easily visible from various measured altitudes and points with the UAV. UAVs can track users in 3D with high mobility. Traditional ground–to–ground communications suffer from higher route loss attenuation and blurring. UAV s can provide a better LoS channel probability. In such situations, a few key areas must be considered. Millimeter waves (mm–wave), for instance, are used in 5G systems. LoS is essential for delivering high recurrent transmission capacity to the network. Since the LoS condition allows for effective beamforming in 3D space, UAVs are good candidates for 3D Multiple Input Multiple Output (MIMO). The idea of using UAVs as BSs is represented in Figure 4.

#### 2.4.2. Normal User

**Figure 5:**
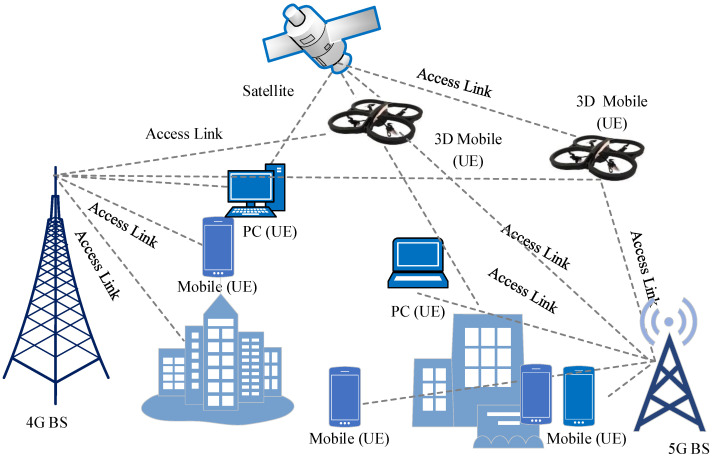
UAVs as normal users in a future ultra–dense heterogenous network.

Due to obstacles in the coordinate LoS path, the signal to and from the BS for a terrestrial UE is regularly deflected or diffracted. As a result, the UE’s gained signal quality will be significantly reduced. BSs are typically located at high elevations, such as cell towers or building tops. The likelihood of obstacles obstructing the LoS path dramatically decreases as the UE ascends to a higher altitude, as in the case of a hovering UAV. The signal quality improves as the path loss decreases since signal propagation through the sky is close to free–space propagation. The UAV can have LoS access to a number of nearby non–serving BSs. The increased likelihood of LoS paths to numerous non–serving cells will increase the UAV’s obstacles since the cells share the same radio assets. The signal–to–interference–plus–noise ratio (SINR) may be low due to the high number of obstacles, making it difficult for the roaming UE to quickly receive and translate adaptable management–related signals (for instance, HO commands). Figure 5 presents the normal user scenario of UAVs in wireless communication.

### 2.5. UAVs in 5G Networks

As commonly known, 5G will transform multiple aspects of society. UAVs will likely be a significant tool used to demonstrate the full potential of 5G technology. UAV connection may even be possible with 4th generation (4G) LTE, which would be advantageous. UAVs are currently used as flying sensors linked to 4G networks. These sensors can convey data over great distances while remaining securely outside the pilot’s line of sight.

The International Telecommunication Union (ITU) provided an overview of the differences between 4G and 5G networks. This agency developed the capabilities that differentiate broadband cellular network generations. When discussing wireless networks, two definitions must be highlighted: upward and Verizon. In 2009, telecommunication operators deployed 4G and continued its management until 2019. During that time, 4G was widely employed, allowing users to download movies and use the GPS in cars. In 2019, Verizon pioneered 5G, launching a commercial 5G ultra–wideband mobile network in different sections of two cities. 5G enables rapid data transmission speeds due to the massive amounts of data acquired from simulating linked devices. Overall, 5G includes high data rates, low latency, energy, cost efficiency, increased system capacity, and widespread device connectivity.

The rate at which data is successfully transmitted across a network is referred to as throughput. Peak data rates of up to 10 Gbps can be achievable with 5G. At this level, driverless vehicles, fabrication, and virtual reality (VR) can rapidly advance. This further indicates that UAVs will be capable of transmitting large volumes of data. 5G technology allows devices to communicate at speeds of up to 500 km/h. Commercial UAVs will be able to inspect vast lengths of highways in minutes while maintaining network connection in such a way that data can be promptly transmitted. The 5G network can service millions of devices in a single square kilometer. Numerous organizations can be completely transformed and developed, ranging from home parcel delivery to search and rescue operations. The energy efficiency of 5G ultra–wideband will also be enhanced. Delays will further reduce, signifying the impact of lower latency. It is not uncommon that audio and visual images lag from time to time. 5G data transmission speeds will be at much faster magnitudes than the blink of an eye, with an end–to–end reaction time of roughly 10 milliseconds. This process provides UAVs and sensor operators with a near–real–time experience. With low latency, autonomous UAVs can navigate with tremendous precision due to instant communication.

## 3. Mobility with UAV Technology

The term “handover” refers to the process of switching from one cell to another while maintaining connectivity. Which is a core part of mobility management, if not the most important part. Beginning with the mobility management and HO concepts, which are regarded as the most important terms for understanding wireless technology in general, this section provides the reader with a comprehensive organized overview of mobility management with UAV technology. Following this conceptual review, we introduce mobility and HO in 3D for a variety of scenarios that correspond to UAV flights through space. This will provide the reader with a solid foundation for understanding the key components of the wireless network infrastructure that supports UAVs [45,46,47].

### 3.1. Mobility Management Concept

In the ideal case, a mobile UE’s connection to the serving wireless network should remain stable even as the UE moves within cells, and this is the definition of mobility in wireless networks. When comparing wireless and wired networks, this is often cited as an advantage of the original. The UE’s mobility allows it to move in a variety of ways. As long as there is coverage, the UE can switch its connection as it moves from the first cell (known as a serving BS) to a new cell (known as a target BS). The original serving BS can reroute the connection to the new target BS. All of these enhancements make wireless services more accessible to more users in more situations. The received signal strength (RSS) fluctuates continuously as the UE moves. A HO procedure is initiated when the RSS at a given location falls below a certain threshold defined by the RSS Indicator (RSSI). First, the serving BS sends a request to the target BSs, requesting that the UE’s connection be rerouted to the target BS with the strongest signal. As a result, in the best–case scenario, the UE’s connection to the serving networks will be stable throughout the user’s journey [45].

### 3.2. Handover Concept

HO is the process of preserving connection in wireless mobile networks, this includes numerous scenarios in which the user maintains a connection while moving from one location to another. The method involves changing the BS that was previously servicing the moving user to one that has a better connection at the moment. This modification occurs through various scientific and technological procedures [45,46,47]. The HO method was developed to manage wireless mobile connections while users are traveling to provide highly dependable and smooth communication. In reality, HO is intended to boost user throughput while decreasing radio link failure (RLF) and interruption time. The dependability and quality of the serving network will improve if the HO management strategy is enhanced [48].

To complete the transfer, three activities must be accomplished. The mobile station may easily locate the surrounding BS in the first phase since the BS broadcasts a mobile neighbor advertisement control (MOB NBR–ADV) that identifies the radio channel and media access control (MAC) address. The target wireless network with HO timing is selected in the second step after scanning for surrounding BSs. Initially, the mobile station sends a scanning request (MOB SCN–REQ). The BS then receives the scanning response (MOB SCN–RSP) to provide a search time to the mobile station, which contains the list of target BSs. The HO decision is initiated by a mobile station HO request (MOB MSHO–REQ). The transition to the new wireless network is completed in the third step. The main HO procedures are listed in Table 1 [35,49].

Several HO methods are used to promote user mobility to define when and how the UE should undergo the process of performing HO. Various optimization options are available, such as selecting the routing protocols and suitable objectives for any access point/base station (AP/BS). The availability of mm–waves in modern wireless technologies (5G and 6G) further complicates the selection of an adequate HO. The latest technologies allow for faster mobility, reaching speeds of up to 350 km/h in 4G and 500 km/h in 5G.

HO is well known for assessing wireless communication performance. Various requirements and indicators were developed to represent network performance during HO operations. The first requirement is that the relationship between the BS and the UE must be kept as stable as possible during the eNB transition. The second requirement is the HO interruption time, which is defined as the time when the UE is not permitted to deliver user plane packets to the BS. To ensure a smooth UE experience, the interruption time should be extremely short, such as less than 1 mms. The third requirement is the HO cost, which is computed by multiplying the mobility interruption time per HO by the number of HOs in the trajectory of a specific UE. The fourth requirement is the HO failure rate, which is calculated as the number of HO failures divided by the number of times the UE processes the HO. The fifth requirement is the signaling overhead, which is defined as data generated during HO processing to simplify the method.

Another issue is load balancing between BS cells. If the cell connection for the same BS is blocked, other UEs would have to move to a different cell. Another benefit of using HO is that it saves money by establishing a connection to a neighboring BS with lower communication power. It further preserves device battery life by regulating the transmitted power [48,50].

### 3.3. Mobility in Three Dimension (3D)

UAVs typically fly at high speeds above the BS antenna height in 3D space. 3D mobility changes the UAV’s altitude which consequently influences the propagation channel characteristics. Thus, 3D coverage that can adapt to changing UAV elevations is required, and speed limitations must be maintained.

#### 3.3.1. Communication Coverage in 3D

The data transmission coverage of a wireless network is referred to as “communication coverage”. When the coverage area shrinks, so does the RSS. The RSS may be defined in 3D space using the altitude value. During the transition phase, the terminal will decide whether to remain on the current network or transfer to an adjacent one as the new base station. The conventional two–dimensional (2D) HO determination approach does not apply to UAVs due to their varied altitude values. To compute the coverage of a UBS, the following equation can be applied [49]:

(1) $$coverage=\pi d^{2}=\pi \left(Radius^{2}-Altitude^{2}\right)$$

(2) $$d=\sqrt{\left(R^{2}-A^{2}\right)}$$

Several strategies and algorithms are used to make HO decisions. The RSS determines the best coverage option, which can be calculated using the following equation [49].

(3) $$RSS_{cur}=RSS_{min}-10\beta \log\left(d\right)+\varepsilon$$

**Figure 6:**
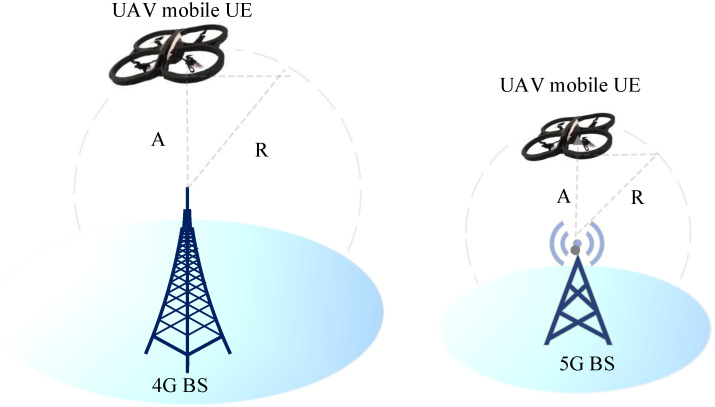
UAV–based station coverage according to altitude. Equations (1) and (2) describe how to produce the *A* and *R*, as shown in the illustration.

The path loss exponent is *RSS_min_*, which is the lowest value necessary for a terminal with a one–meter distance between the sender and receiver, *d* is the distance between the receiver and transmitter, and ε is a zero–standard–deviation Gaussian random variable. Figure 6 presents the radius of the BS, where *A* indicates the height of the UAV and *d* represents the radius of the BS’s coverage in 3D space. HO occurs when the link between the BS and the UAV has stretched beyond its coverage.

#### 3.3.2. Speed Limitation in 3D

Today, WiFi, WiMAX, and cellular technologies are now available networks. Smart devices rely on mobility–based network services, thereby increasing their demand. Consumers expect an internet connection at all times and from any location. It is important to remember that when the UAVs are traveling faster than the UE, HO may frequently occur. HO depletes energy and causes connection delays. To solve this issue, the speed of the UAV must be limited by using the following equation:

(4) $$Speed\;limit\left(\alpha \right)=S\left(\frac{w}{\delta }\right)$$

where *w* signifies the RSS value. As the distance between the BS and the terminal increases, the received signal intensity decreases. The scenario then moves to the execution phase when the RSS falls below the threshold [49,51,52].

### 3.4. Handover in UAV Networks

HO performance is a crucial indicator of the UAV network performance since it possesses a level of network flexibility. UAV coverage fluctuates based on transmission power and altitude. Researchers are investigating seamless HO to provide ground users with dependable HO. Traditional cellular networks and HO in UAV networks are not similar. To deliver ongoing services to mobile users, an intelligent HO approach has been devised. Effective solutions based on enhanced software have been provided to achieve quick HO in UAV networks. Knowing the quantitative expression of the likelihood of HO can aid in the construction of system gridlines. Small stochastic geometric models in UAV networks can also be created for evaluating mobility performance. By simulating UAV movement with a random mobility model, the statistical aspects of the channel gain can be examined [53,54,55].

### 3.5. UAV Handover Scenarios

In 3D space, UAVs typically fly much faster than the average BS. Additionally, the UAV can function as either a standard mobile UE or a fly BS. Different types of HO scenarios emerge for two cases based on these arguments.

#### 3.5.1. HO Scenarios with Flying Base Stations

**Figure 7:**
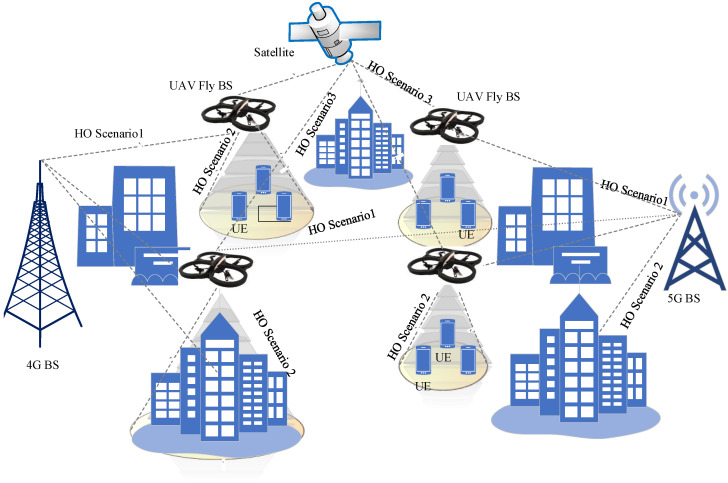
HO for UAVs as base stations in a future ultra–dense heterogenous network.

When a UAV is used as a fly BS, three scenarios can exist. In the first possible scenario, the UAV experiences HO when it changes its ground BS. In the second type of HO scenario, the UE changes from connecting to the serving UAV BS to connecting to another serving BS. In the third type of HO scenario, the UAV experiences HO when it changes its serving satellite node to another one. These scenarios are illustrated in Figure 7.

#### 3.5.2. HO Scenarios with Normal User

**Figure 8:**
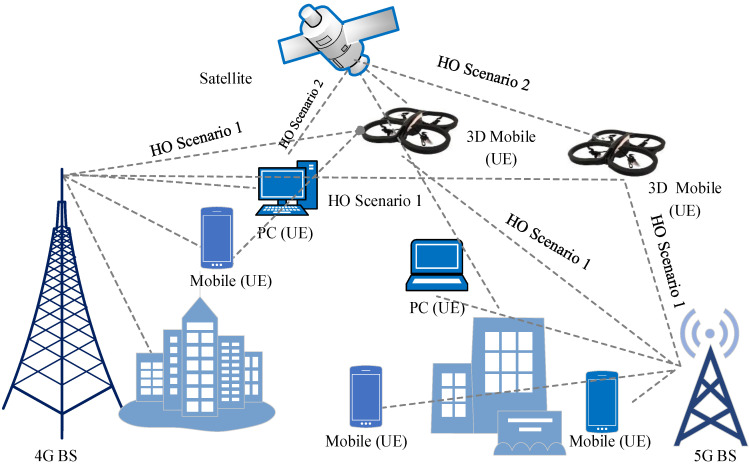
HO for UAVs as normal users in a future ultra–dense heterogenous network.

The UAV acts as a mobile user above the ground, and there will be two possible scenarios involving the UAV changing its connection to a different BS. This is also possible for satellite communication systems, as evidenced by the fact that UAVs can switch from using one satellite node to another. These scenarios are illustrated in Figure 8.

### 3.6. UAV Handover Based on Machine/Deep Learning

In 5G networks, the use of mm waves with higher frequencies will present new challenges for HO management, which will be difficult to overcome using traditional methods. Significant attenuation in these frequency ranges will initially be present, limiting their transmission distance. As a result, more BSs are required to cover the same area as those using microwave frequencies [56]. Directional beams are used in mm–wave transmission. Obstacles in the path of the transmitted beam may prevent the user from connecting to the network or deteriorate signal quality. As a result, users in mm–wave communication networks must determine which beam to connect to at any given time to optimize their QoS. Deciding on the best beam has become a new factor to consider in the HO management process. The large number of beams which the user must choose from makes the HO technique significantly more difficult [57,58,59,60].

The network’s self–optimization will improve with the use of machine learning techniques. ML techniques can learn diverse attributes from data provided by the network to maximize different network sections. They can detect hidden network features and patterns in network data that analytical methods are unable to detect [61]. They are self–adaptive, which means they can respond to changes in the network environment and, in some cases, anticipate future organizational or user needs. This allows the network to prepare for them when they occur [62]. They can be written in such a way that the preparatory stage of the calculation, which is often computationally expensive, can be completed offline before moving on to the actual calculation [47]. The planned program is broadcast online to allow for real–time optimization, yet the model is rarely updated since it encounters unnecessary data [63].

## 4. Research Challenges

UAVs–based networks face numerous challenges. It is difficult to establish reliable, low–latency UAV control communication in a cellular network. The availability of infrastructure is a major goal for improving terrestrial communication services. Terrains with limited terrestrial BS coverage may be unable to provide connectivity services to cellular–connected UAVs, necessitating a solution for effective deployment of the technology. Numerous studies and research projects have demonstrated that low–altitude UAVs can be used in conjunction with cellular networks. With low–latency, high–throughput cellular network spectrums, UAV integration will be possible. According to 3GPP, aerial UAVs have lower SINR than terrestrial UEs. Excessive HO failures and HOs are among the issues. These concerns must be addressed because the consequences could seriously impact network stability in the future. Consider environmental implications, routing protocols, channel effects, antenna designs, and HO management to maximize UAV benefits. These issues must be addressed to properly connect UAVs. A UAV–based cellular network faces challenges in terms of 3D coverage area and ground channel [36,64,65,]. These challenges are discussed in this section and summarized in Table 2 so that the reader can get a complete picture of them.

### 4.1. General Challenges of Connected UAVs

One of the main concerns is the dangers associated with monitoring airborne applications. Pilot preparation, flight length, climate conditions, and risk restrictions are all key factors when transporting a tracked flying machine [66,67]. Connected UAVs and unmanned aerial system (UAS) technology can be used to place unmanned airships in situations where a human pilot cannot be placed due to various risks. UASs can also be used to gather extensive information regarding the progress of human activities to aid in risk mitigation and reduce the amount of time people spend in potentially dangerous situations. UAS is fully embraced by several development organizations to help mitigate the risks associated with various situations, such as foundation reviews, for instance. The vast majority of assessments rely on ‘human eyes’ on the ground to inspect the condition of basic components and determine whether or not maintenance is required. Support groups can evaluate the framework with low–cost UASs while staying on the ground, avoiding dangerous and time–consuming hikes. Take, for example, how energy firms assess the major arch framework [68]. On rare occasions, administrators may choose a helicopter inspection or require a support crew to attempt a climb to examine the arch foundation from the outside. Maintenance personnel can use UAS to conduct an initial inspection from the ground, avoiding perilous climbs and reducing casualties. Due to the fact that a UAS inspection takes less time and requires fewer people than an actual climb, groups can inspect arches more frequently or with a smaller team. If an irregularity is discovered, its severity and impact can be assessed on the spot. Although a human climb may be required to solve the problem, the maintenance crew can ensure that they have the replacement components, the right instruments, and the right people on hand to fix and complete repairs. Individuals can also be rescued from dangerous situations and accidents by implementing comparative risk reduction and mitigation strategies across numerous verticals [69,70,71,72].

### 4.2. UAV Operations in LTE

Long Term Evolution (LTE) is well–suited to serve aerial vehicles, such as UAVs. Field tests where LTE systems are used to connect UAVs to networks are becoming more common. UAVs are expected to rapidly grow, providing modern and exciting trade opportunities for new LTE telecommunication companies. LTE network enhancements can be made in the near future to better prepare for the expected increase in data traffic from aerial vehicles. Radio propagation parameters encountered by an airborne UE, for instance, are likely to differ from those encountered by a ground–based UE. The behavior of an aerial vehicle is normal as long as it flies at low altitudes to the BS radio line. After flying well above the BS antenna height, the UL signal from an aerial vehicle becomes more visible to many cells due to LoS propagation conditions. An aerial aircraft’s UL signal can block the cells around it. Increased interruptions are harmful to UEs on the ground, such as smartphones and IoT devices. To maintain normal throughput performance for the UE, the network may impose restrictions on the admission of aerial vehicles within the organization. UAVs also have administrative authorizations that are unique to them. There are two types of “UAV UE” in the field. The first is the UAV with a cellular module that is approved for use in the air. The second is the UAV with a cellular module that is only authorized for terrestrial use. Not all districts allow their usage due to administrative concerns since the UL signal from a UE can block nearby cells. Another point to emphasize here is that the processing time in LTE systems is high, and the mobile station is moving quickly, which may raise the possibility that LTE will not be a supportive network for UAVs, particularly in high–speed scenarios. In addition, the HO delay will be extended beyond the standard 30 ms execution time. This may cause the UAV to fly outside of its coverage area without performing the necessary HO, disrupting the connection and reducing communication efficiency. Because LTE networks have a limited capacity and BW when compared to 5G and 6G networks, they may not be suitable for supporting UAV communication due to the massive growth of mobile devices connected to the network.

### 4.3. Mobility in 3D

Current radio access technologies are not well suited to promote flying radio devices since their formations are largely geared to assist terrestrial UEs. BSs are typically built and modified to provide the best possible performance for ground users. Existing BSs have been modified to achieve the aforementioned goal. The downward tilting antennas produce radiation patterns that are unsuitable for serving aerial UEs, which are expected to be positioned at various heights above ground level. Since the aerial user frequently flies above the BS antenna height, 3D coverage that can adapt to changing UAV elevations is required. The BS antennas of LTE networks may be able to achieve efficient channel gain by utilizing their side lobe antennas. The BS antenna length, UAV height, antenna design, and association criteria all play a crucial role in determining UAV coverage patterns in 3D space. As a result, a 3D coverage model for aerial users corresponding with terrestrial users is required for the network model. UAVs for network services are different from traditional networks in that they use a 3D model rather than a 2D model to create mobility. UAVs are extremely mobile, rendering their control and decision–making processes difficult. Advanced mobility solutions will be required as a result [49,73,74,75,76].

### 4.4. UAV to Ground Channel

Creating coexisting mechanisms between terrestrial and airborne users is one of the most complex design challenges for developing cellular–connected UAVs. To achieve this coexistence, UAV–ground interference management must be installed. Unlike the ground BS to ground UE communication link, the ground BS to UAV communication link has very different interruption patterns. UAVs may establish LoS communications that are more dependable than those with terrestrial users since they fly higher than BSs. They also make use of significant macro–diversity gains provided by many BSs. Ground users, on the other hand, generate more UL/DL interference than the dominant LoS connections, making Inter–Cell Interference Coordination (ICIC) extremely difficult. Fading, shadowing, and route loss are also important considerations. Traditional ICIC solutions may be adequate for existing cellular designs; however, they fall short when it comes to UAV interference control which involves a large number of BSs, imposing limitations due to its complexity will emerge. As a result, effective interference management strategies are required for the coexistence of ground users and UAVs. Several books on the subject of downlink and UL up–link interference are available [49,77,78,79].

The most common types of links in the communication channel are Ground–to–UAV (G2U) and UAV–to–Ground (U2G). The G2U link provides downlink control and command for suitable UAV operations in cellular–connected UAVs, while the U2G link provides UL payload communication. Rayleigh fading is the most frequently used small–scale fading model for terrestrial channels, however, Nakagamim and Rician small–scale fading are more common for U2G channels due to the presence of LoS propagation characteristics. Large–scale fading is altered due to the 3D coverage area and the varying heights of UAVs. A free–space channel model, an altitude/angle–dependent channel model, or probabilistic LoS models can be used as large–scale fading models, as follows:

1. In the free–space channel model, fading and shadowing have little effect and interference is low. This method is most effective in areas where the LoS assumption holds true between high–altitude UAV s and ground stations. Low–altitude UAVs may encounter non–LoS connections in urban environments, necessitating the use of additional methods to accurately assess the propagation environment.
2. In altitude/angle–dependent channel models, channel characteristics, such as shadowing and path loss exponents, are affected by the UAV’s elevation or angle. Depending on the deployment, these varieties can be used in residential or sub–residential settings. Altitude–dependent models may not be appropriate if the height does not change or if UAVs fly horizontally. In analytical research, models based on elevation angles are commonly applied, but there is insufficient literature on the subject.
3. Due to buildings, obstructions, or bottlenecks, approaches based on probabilistic LoS models are frequently allowed for residential scenarios where the LoS and NLoS links between UAVs and the ground are recognized. The LoS and NLoS components are separately displayed according to their likelihood of occurrence in a home environment. Their characteristics propagation are statistically determined by the nature of the residential environment in terms of building height and density.

### 4.5. Transmission Protocols

Data in UAV–based connected networks must be rerouted from one serving UAV BS to another serving BS because UEs switch from one BS to another during the HO process. This is an important point that makes the transmission protocol important in UAV–based connected networks and should be highlighted. Several companies have successfully filed patent applications detailing how UAVs to scan and acquire data while dropping data packets, such as a queuing delay and transmission delay (QDTD) routing protocol. This solution, on the other hand, employs a complex processing method that necessitates more computation time, resulting in data delays and a decrease in data throughput [80]. Another protocol is an adaptation of the distance routing effect algorithm for mobility (DREAM) protocol, which includes a location service, a local database, and a routing agent. The location service keeps track of each node’s location. To compute location, the distance effect given by the difference in velocities of two nodes is used [81]. The Media Access Control (MAC) layer is another protocol, and directional antennas are used at the top and bottom of the aircraft. The UAV detects the medium to determine whether or not there is any active communication [82]. TCP/IP and other traditional transmission methods will be insufficient for UAVs. As a result, new procedures based on the mobility characteristics of UAVs must be developed [83].

### 4.6. Dominance of LoS

The radio environment differs from the terrestrial environment; therefore, issues may arise as elevation increases. UAVs cause significant disruptions to BSs in cellular networks when aerial and terrestrial users work together. This is a problem for terrestrial users who utilize UL communication services. The prevalent LoS determines the characteristics of UAV communication channels. They will have an unavoidable impact on the HO mechanism since no barriers are present in the sky. This characteristic must be considered when constructing UAV–based networks. UAVs are subjected to frequent HOs and ping–pong effects as a result of their fast mobility, which causes rapid channel shifts. Several studies have suggested methods for reducing UAV crashes in cities while simultaneously easing traffic congestion. UAVs may encounter unexpected scenarios or tasks in smart cities which require relevant solutions. A number of drawbacks must be addressed in HO research for UAVs [84,85].

## 5. Related Works

The literature is crucial when it comes to incorporating UAVs into future networks. This section includes a review of relevant studies. Several research techniques, as well as the findings and outcomes of these efforts, are briefly described along with suggestions for future improvements. These papers mostly discuss UAV HO decision algorithms based on mathematical models or machine learning techniques. UAV architectures and new use cases are also examined. The following research papers are listed in chronological order and a summary of challenges and previous contributions has been provided in Table 2 and Table 3. This overview can provide insight into the best UAV integration techniques and serve as inspiration for future efforts.

UAVs and cellular networks are becoming increasingly popular research topics. Recent proposals with unique solutions have been made to tackle scientific, technical, socioeconomic, and security issues. Several surveys, examples, and tutorials are also presented in the literature to provide clear information regarding this research topic. These works allow the research community to keep track of ongoing studies, and aids practitioners and researchers in acquiring necessary information. Several surveys and tutorials have focused on (a) the possibility of integrating UAVs with 5G/B5G cellular networks from the aspect of UAV–based cellular communication, (b) current advances, future trends, and challenges for UAV–based cellular communication, and (c) extensive analysis and performance studies regarding a specific communication challenge, such as channel modification.

The authors have presented a method for determining a UAV network’s coverage. Constraints in the UAV network, battery capacity, and HO management have led to communication disruptions and other challenges, such as regular HOs. Since UAVs are positioned at different coverage areas and heights, traditional HO algorithms do not work. To maintain coverage, the recommended solution uses RSS to change the height and separation distance of each UAV. Several simulations were accomplished to determine the likelihood of a smooth transition using seamless HO success probability (Ps) and false HO initiation probability (Pf). Since the coverage algorithm matches all UAV heights to a value (the lowest coverage and heights to the lowest feasible value), the spacing between the UAVs can be modified using Pf and Ps. Pf grows as the vertical space between the overlapping sections shrinks. These sections shrink as the distance between the UAVs increases and Ps decreases. The chances of achieving a smooth HO decrease as the average RSS measurement increases. The chances of an inaccurate HO begin to lessen as the average RSS measurement duration increases. According to the simulation results, the proposed technique is a strong candidate for UAV networks. The method performs admirably when it comes to simulations. However, a more realistic scenario must be considered, such as the UAV’s payload, the radio range of the BS, and other factors. Moreover, the coverage algorithm equalizes the RSS of each UAV, which may or may not be acceptable in practice.

In 2004, 2010, and 2012 [86,87,88], a novel HO decision technique was developed by establishing innovative HO criteria. The HO decision was made using a fuzzy inference method that considers several factors in HO decision situations. This paper examined fuzzy MADA methods and various proposed methods based on this approach, as well as their sensitivity. The HO approach based on an optimization algorithm was also suggested for cellular networks.

In 2004, 2012, and 2016 [88,89,90], the HO method was discussed for 3D aerial networks. As we know, the 3D method differs from the classic 2D approach. The height of the UAV and the distance between UAVs must be adjusted. The likelihood of seamless successful HO and false HO was also evaluated for the best coverage assessment technique. The authors devised the HO decision to select the appropriate network. The use of a fuzzy logic approach led to the ability to manage inaccurate data, which is a useful enhancement to this approach. This enables it to make multi–criteria decisions. The authors then created an adaptive HO management approach based on a fuzzy logic system that works in conjunction with an existing cross–layer HO protocol. Based on the compression between the performance of both the existing and proposed approaches, the suggested technique outperforms the traditional method with noticeable intra–system and inter–system HO.

In 2007, 2008, and 2014 [91,92,93], MATLAB was employed to develop a vertical HO scheme. Since MATLAB was the platform used, the proposed approach is suitable for wireless wide area networks (WWAN) and cellular networks. To construct the fuzzy logic quantitative decision algorithm (FQDA), eight factors were considered. There were 81 rules used for the eight factors, which were then compared to 6561 rules. Algorithm models were developed and implemented according to various criteria based on vertical HO. These vertical HO methods were demonstrated in the heterogeneous wireless networks (HetNets) WWAN and WLAN environments. The methods are per the IP–based workforce automation sector, which allows unrestricted mobility across networks while connecting via IP mode using a single device on multiple networks. The vertical HO approach was used to integrate Wi–Fi (IEEE 802.11) and WiMAX. The signal–to–noise ratio, moving speed, and signal strength were all factors considered in this study. NS2 and NS3 were used to create the simulation.

In 2010, 2013, and 2019 [94,95,96], two approaches to vertical HO were introduced in a HetNets environment. The fuzzy interface system was used, as well as subtractive clustering techniques. According to the simulation, the approach enables the HO procedure to become easier and faster for different protocol users. The authors proposed a method for conserving energy and battery life by using fuzzy logic. Mobile phones with LTE and Wi–Fi capabilities can also be useful in reducing battery consumption. Researchers proposed a method for performing HO in 3D space by considering speed and coverage constraints. A fuzzy interaction system was created to make HO decisions.

In 2010 and 2014 [35,97], a speed adaptive system with a knowledge method was created to enhance the rate of the network’s candidate set. The decision algorithm was developed as a collaboration between vertical handoff, fuzzy logic, and pre–HO decision in order to generate effective and efficient judgments. A performance study was conducted to compare the proposed work with the typical RSS. According to its findings, the suggested method improved performance in terms of reducing unnecessary HO and the rate of call blocking or dropped calls. Many HO algorithms were measured and used to reduce HO in the network. According to the results of this survey, popular algorithms were developed to address complex challenges, which sometimes lack clarity or sufficient detail.

In 2011 and 2018 [56,98,99], the authors listed several problems that mobile aerial users face. In most recent static cellular deployments, the sidelobes of the antenna design assist aerial users. As a result, the connection pattern is broken. Due to the fragmented connection and low SINR, a higher risk of radio connection and HO failures are present. The uneven connection pattern, in which a user is returned to its original cell within a set time limit, will lead to more ping–pong HOs. While LTE is designed to allow users to travel at speeds of up to 350 km/h, it is based on large cell areas rather than the sidelobe–based cell attachment patterns seen in UAVs. The 3GPP research item has identified cell selection, HO efficiency, and robustness as critical performance criteria for aerial users in cellular networks.

In 2013, 2015, and 2016 [75,100,101], the authors implemented a machine learning technique for the UAV network as a potential solution. The machine learning technique is seen as a promising approach in this field since it can predict node mobility. Currently, prediction solutions are based on distance measurements. To address a two–dimensional issue, a categorization of movement to other classes based on nodes’ prediction of near future positions has been proposed. Acceleration also has a significant impact on the likelihood of 3D node movement. The motion trajectory was calculated using state transition equations to determine the object class. The calculations were then used to clarify the mobility parameters. To complete these procedures, several steps must be accomplished. The most important step is to use an online class identification module to determine the classes and parameters that were unspecified but acquired from observed trajectories, while keeping in mind that each UAV has its tracking system, including Automatic Dependent Surveillance–Broadcast (ADS–B) technology and GPS positioning. The Kalman filter was used to achieve 91% accuracy in motion profiling. The online module generated more classes over time. Kalman filtering with intermittent observation forms the backbone of this approach, allowing for simultaneous estimation of the target vehicle’s position, velocity, and acceleration using the relative position and velocity information provided by the radar system. Kalman filtering, which contains two sets of time updates, can be used to solve the state transition equations and obtain a reliable approximation of the state vector. The next state vector (position of flying item) is predicted using time update equations. Time update equations are used exclusively when no measurement is available to predict in the case of intermittent observation. The optimal state estimate of singular systems provides a solution for this system given an unknown input.

In 2016–2021 [28,47], the authors proposed an efficient HO mechanism for UAV networks. UAV network services differ from typical networks since the HO process is carried out in 3D rather than 2D. To enhance network services, this technology adjusts the height and distance between UAVs. The ideal coverage selection technique is assessed using the seamless HO probability and Pf. To ensure that each UAV covers the same area, the height of each UAV must be adjusted to account for physical limitations. A seamless HO is possible in certain circumstances. Ps and Pf have been modeled in numerous scenarios to examine how they change. A large number of graphs were obtained for investigation and evaluation. The vertical distance between the overlapping sections becomes smaller as Pf becomes higher. The overlapping area shrinks as Ps shrinks. Overall, this technique can help UAVs preserve the environment. The battery can last longer by avoiding frequent HOs. The proposed method can assist in optimizing a UAV network by determining the ideal overlapping region. By assigning the same RSS to all UAVs, UAV interference can be reduced. Although the study did outline the preferred method, several factors must be considered. The most serious issue is that adequate coverage for UAVs is difficult to achieve. If an obstacle prevents the UAV from flying to a lower altitude, for instance, the UAV’s minimum height must be adjusted. The RSS level for moving UAVs must be raised to maintain smooth HO when the UAV is influenced by weather factors, such as wind. It is also necessary to consider the system’s dependability and throughput rate.

In 2018–2019 [99,102], the authors realize that providers cannot sacrifice ground–level performance for aerial users by changing the BS antenna angle. According to several studies, the BS will be able to spatially separate users in 3D space by using directed antennas and beamforming, allowing for effective service of both ground and aerial users. Experts believe 5G is a good choice since it allows beamforming and high throughput connections while remaining significantly flexible.

In 2018 and 2021 [47,103], it was found that UAVs are especially vulnerable to LoS propagation, which is required for mm–wave communications to work. Use of mm–wave communications was suggested as a possible option. Larger path loss reduces inter–cell interference for mm–wave frequencies, while small antenna aperture size allows a large number of antennas to be used in the antenna array. Arrays can be employed to provide beamforming which compensates for the user’s high path loss while simultaneously reducing interference. The application of mm–waves opens up a significant usage spectrum. The high throughput previously mentioned can be easily achieved by using a large bandwidth.

In 2018, 2019, and 2020 [104,105,106], the authors used simulations to further investigate this problem, discovering two issues. The first issue is that high levels of interference will make it impossible to maintain connection and complete successful HOs, resulting in a high percentage of radio link and HO failures. The focus shifted to LTE–M, a technology that allows users to communicate in a low SINR. The authors were able to reduce the number of radio connection and HO failures by simply increasing the number of ping–pong HOs. The second issue is that the default HO strategy will fail when aerial users transmit antenna pattern nulls. The volume must be kept low to avoid a radio connection failure. Fine–tuning parameters of the HO mechanism, such as the reaction time, was suggested to solve this problem. The introduction of 5G networks will alter people’s communication habits. Several tests were conducted with a UAV connected to a 5G BS at a frequency of sub–6 GHz. HO to the 4G network automatically occurred. The UAVs experienced more HOs than land users, lowering the overall throughput. Researchers believe this will be corrected with the deployment of more 5G BSs.

In 2019–2020 [107,108], the authors considered equipping UAVs with highly directional antennas. They suggested the use of 5G’s massive MIMO capabilities since it allows the BS to geographically separate users while simultaneously producing nulls for other users to prevent interference.

In 2021 [109], the authors mainly focused on static users, however, new issues emerge when mobile circumstances are considered. Beam training and tracking become more difficult, resulting in significant amounts of overhead. However, this overhead is lower than expected in the simulations, allowing mobile users to be serviced at standard rates. Another issue with using mm–waves is the large Doppler frequency changes that are proportional to the center frequency.

**Table 3:** Summary of previous contributions.

No	Author	Contribution	Limitations\Research Areas
1	Azari	To overcome the HO and Radio Resource Management (H–RRM) problem, a deep reinforcement learning approach was developed [84].	It concentrated on UAV as a user while ignoring UAV implementation as fly BS.
2	Yun Chen	A unique HO framework was offered to provide competent mobility support and a reliable wireless network to UAVs that are supported by a terrestrial cellular network. A deep Q–learning strategy was created to powerfully optimize HO decisions, ensuring a robust network for UAV users using instruments from deep reinforcement learning [110].	It did not address the inclusion of 3D UAV mobility in the present framework.
3	Park et al.	A coverage choice method was presented for UAV networks. UAV network restrictions, such as battery capacity and HO management, have caused faulty communication and other issues, such as frequent HOs [78].	It restricts the key points on UAV height while ignoring all other aspects.
4	Park et al.	As a continuation of their previous work, an efficient HO mechanism for UAV networks was proposed. Since the HO mechanism is accomplished in 3D rather than 2D, the network services of UAVs differ from traditional networks [79].	It used RSS as the key point, but in practice, the RSS value may vary with LoS and NLoS, thus another metric, such as SINR, should be considered.
5	Mangina et al.	A system that combines an unmanned semi–autonomous quad rotor with a VR–based scheme was presented [111].	Experiments are limited by labs, so the challenge is to use UAVs as a UE to make assistive technology work better in the real world.
6	Bae	Using UAV telepresence is a powerful tool that many people may take advantage of. Existing robot technologies, on the other hand, are largely for indoor use since their mobility is sometimes difficult and problematic [112].	It needs additional development to minimize weight and increase power consumption efficiency. Furthermore, the tests must imitate real–world conditions.
7	Orsino et al.	A simulation was suggested to investigate the implications of HetNets mobility on Device–To–Device (D2D) and UAV–assisted Mission–critical machine–type communications (mcMTC) in 5G [113].	The heterogeneity of the equipment employed, such as UAVs, Fiber, and masts, causes operational challenges that must be handled by the quickly expanding industrial IoT ecosystem.
8	Lee et al.	A fuzzy inference method was used to create an intelligent HO scheme for UAVs. The system makes HO decisions via a fuzzy inference process [49].	Look at approaches to improve the functions of the HO decision for a variety of devices, including both UAV scenarios as fly BS and UE.
9	Peng et al.	A cutting–edge machine learning method was offered to address the issues arising from UAV network requirements [75].	Unsupervised learning from raw data is a time–consuming procedure.
10	Sharma et al.	The Ultra–Dense Cloud–UAV Network architecture (UDCUN) was suggested [114].	A small coverage area means two cells may overlap, causing co–channel interference. More users near user–site APs make HO regulation difficult without too much communication expense and latency.
11	Yoo et al.	The UAV Delivery Using Autonomous Mobility (UDAM) idea was presented for delivery services. Nowadays, people use E–commerce for nearly everything [105].	Limited evaluators from limited companies evaluated the proposal, limiting the research’s scope. No existing notions were compared numerically.
12	Hu et al.	A deep learning–based system for trajectory prediction and an intelligent HO control approach was presented for UAV cellular networks [115].	Deep learning’s predictive power demonstrates its future utility. However, various challenges must be addressed, including spectrum, energy, and security management.
13	Nithin	A location module was built to improve Over–The–Top (OTT) application location services [116].	Advanced machine learning could enable address discovery, navigation, and product delivery in the future.
14	Guan et al.	The use of mm–waves and Terahertz (THz) band communications in UAV networks were examined where the transmitter and receiver are both mobile [117]	Beam alignment frequency and directivity angle control in mm–wave/THz bands for studying mobility and weather conditions can be future research topics.
15	Euler et al.	The effects of changing radio environments and complications regarding UAV performance were analyzed [104].	To improve the results, future studies may explore avoiding low SNR sites and using directional antennas for the UEs.
16	Banagar et al.	A stochastic geometry–based UAV cellular network model was assessed. Lately, UBSs have been receiving significant attention due to their versatility and wide–ranging applications [118].	Future work will focus on the mathematical analysis of complex mobility models like Random Waypoint (RWP) and Random Walk (RW).
17	Fakhreddine et al.	An experiment in a suburban setting was proposed to see how parameters influence cell selection and HO management when UAVs are employed as aerial UEs [85].	Connecting a UAV to a cell–based solely on the RSRP value and ignoring other key point values like SINR.
18	Banagar et al.	For UBS networks, a stochastic geometry–based mobility model was developed. The mobility of wireless nodes has a significant impact on the performance of wireless networks [76].	The flying BS that served ground UE was restricted to a constant height. A dynamic height may be proposed in the future to reflect the real 3D movement of UAVs.
19	Iranmanesh et al.	A Delay–Tolerant Network (DTN) technique was suggested for UAV communication packet routing optimization [83].	The work discussed UAV issues and offered graphics to illustrate the conclusions while employing a unique packet–based technique. However, future improvements to this algorithm or others are possible.
20	Bai et al.	A new approach (dubbed the route–aware HO algorithm) was suggested to improve UAV communication system reliability [119].	Improved estimation accuracy and granularity in presenting radio link quality can improve the findings even further.
21	Amer et al.	The probability of coverage and the impact of various parameters on the overall performance of the proposed system were examined [120].	Although main and secondary lobes are used to evaluate antenna layouts, side lobes and nulls have an impact on UAV–UE cell allocation and HO in practice.
22	Azari et al.	A machine learning–based technique was recommended for the HO mechanism and resource management of cellular–connected UAVs. When aerial and terrestrial users coexist in cellular networks, UAVs create significant interference to BSs, posing difficulty for terrestrial users’ UL communication service [84].	More DL work is needed to make this study’s results relevant in the future.

## 6. Proposed Solutions

**Figure 9:**
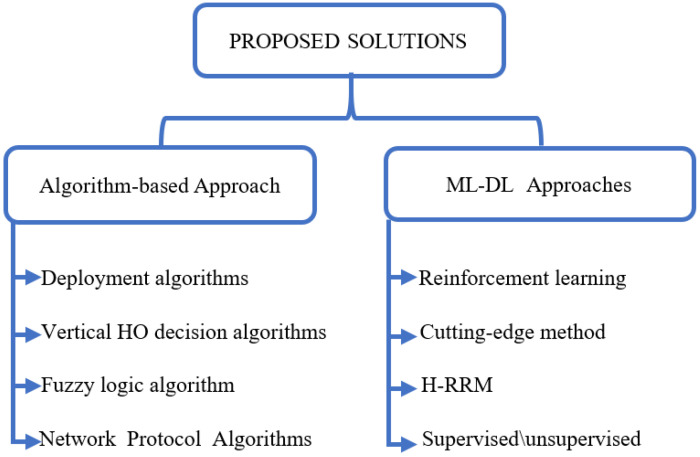
To provide a complete picture, the main classification of proposed solutions can be illustrated as two main categories.

With the increase in connected devices and related services, concerns have emerged regarding mobility and connection. Several configurations have been suggested throughout the literature. In the following subsections, the most common configurations are discussed. The configurations are organized according to the problem that must be solved and the method that will be used to solve it. Figure 9 demonstrates the classification of the proposed solution.

### 6.1. RSS–Based Algorithms

RSS data is used in algorithm–based HO management systems. RSS–based computations are generally less complex, but they are also less precise. Calculations have the benefit of allowing multiple factors to be considered in the HO decision–making process. This decreases computation complexity while further improving efficiency and precision. A method based on RSS was proposed to adjust the altitude of the UAVs and the distance between them using Ps and Pf to evaluate the optimum computation range. To increase the UAVs’ scope to the same level, the height of each UAV can be adjusted while considering the physical constraints. This method is also RSS–based. It manages the range of each UAV by adjusting the height and distance between them. The Ps and Pf are calculated to evaluate the suggested configuration [78,83].

### 6.2. Route–Aware HO Algorithm

The route–aware HO algorithm was proposed to make use of path data. The data from flight paths is used to optimize the network, reducing the number of unnecessary HOs and the likelihood of an incorrect HO. The airborne channels’ consistency and pre–determined directions are applied to manage flexibility. In addition to the offline–based calculation, an online–based calculation was presented in which HO is triggered as a result of SINR computation. The final option entails setting updates regularly. As a result, it can reduce computation complexity while speeding up the execution of active wireless systems [63].

### 6.3. Delay–Tolerant Networking (DTN) Algorithm

A novel concept known as the DTN method (also known as Weighted Flight Path Planning (WFPP)) was proposed to maximize packet steering in UAV communication. The weight of packets is determined by their requirements, the time they must survive, and the amount of electricity they may consume. If the UAV’s maximum length is less than the maximum length it can fly, the method generates an unused path that can be used. The path is obliterated if this is not the case [83].

### 6.4. Machine/Deep Learning Approaches

In recent years, machine learning and deep learning–based methods have been at the forefront of research. Thanks to advancements in the field of artificial intelligence, these ideas can ensure progress in HO decision–making, simultaneously reducing computational costs and addressing security concerns. Since information designs do not require frequent overhauls, the precision and effectiveness of asset utilization can be improved.

In [84,112], the UE’s movement properties are recorded using a hidden layer. Social pooling is also used to capture the interaction between UEs. The four essential activities applied to complete HO are estimation, detailing, judgment, and execution. Unlike the standard HO, machine learning is employed to predict future trends. A confirmation method determines whether the customer should be transferred to another ABS. The optimization issues (HO and H–RRM) are defined by machine learning arrangement strategies that aim to capture relationships at worldly and spatial levels to create an appropriate HO choice. The buffer line is used to characterize the information entry rate, the apportioned range, and the impedances from BSs. Communication of the demonstrated framework is through the air–to–ground channel where the LoS path prevails. The optimization problem is then created, and the results are used to finish the decision–making process and remodel the HOs.

## 7. Future Research Directions

Despite the potential of combining UAVs with 5G methods, research into UAV–assisted wireless networks is still in its infancy. Several unanswered questions must be further investigated. This section highlights the most explored topics for future directions in the field.

### 7.1. Mobility Management

In future HetNets, managing UAV mobility will be a critical factor that requires thorough investigation. Due to their development features, UAV mobility poses a great risk since they rapidly move in 3D. The use of mm–wave groups in 5G and 6G systems is also a significant issue that adds to UAV mobility challenges. The massive expansion of UAVs and mobile connections will further create new problems since mass adjustment will be a significant task that necessitates a productive arrangement. The mobility management of linked UAVs must be properly addressed in future systems.

### 7.2. Energy Charging Efficiency

Energy constraints are a significant obstacle in any UAV communication scenario. Subsequent advancements in battery technology, such as improved lithium–ion batteries and hydrogen fuel cells, have enhanced energy charging to extend flight durations by using renewable energy sources such as solar power. The efficacy of energy charging, however, is significantly reduced due to longer removal time and irregular energy access. To improve charging productivity, novel energy transmission enhancements (such as energy beamforming using multi–antenna techniques and dispersed multi–point wireless power transfer (WPT)) are of great interest. The more important point to emphasize here is that addressing mobility management will improve power consumption efficiency. The goal of UAV mobility management is to reduce unnecessary HO processes, which in turn reduces the HO rate and head over signaling, saving more power and increasing energy efficiency.

### 7.3. UAV–to–UAV and Satellite–to–UAV Communication

When using a UAV as a communication terminal, the Doppler effect, pointing error effect, and atmospheric turbulence effect should all be carefully considered. To receive the frequency–shifted optical signals caused by the Doppler effect, the bandwidth of the optical filter at the receiver should be increased. When analyzing UAV–satellite channels, attention should be paid to the optimization effect in terms of cost efficiency. The receiver diameter design is related to the payload of a UAV in a DL and the restrictions on a satellite in a UL, whereas the transmission power design is related to the payload of a UAV in a UL. Because of practical effects such as the Doppler effect, atmospheric turbulence, and pointing error are all considered. It is also important to note that a swarm of UAVs forms a multi–hop network that assists ground wireless devices in transmitting and receiving packets, each of which contains a direction, in order to provide communication services over a relatively large area. Due to the high–speed flexibility and the need to maintain close communication links with ground users, the interface connection with nearby UAVs is disengaged as much as possible. All standard steering protocols will not work with FANETs in this scenario. As a result, mastering UAV flight control may be difficult. When multiple UAVs collaborate, avoiding collisions becomes a critical issue for UAV security. As a result, point–by–point proliferation sequences are required in modern satellite–to–UAV channel models [121].

### 7.4. Interaction between Different Segments

Using new methods to provide continuous integration between space–based networks, air–based networks, and the ground cellular network is a key challenge for the integrated space–air–ground network. It is crucial to incorporate several key factors into various cases. Cross–layer convention plans are required to ensure interface consistency. It is also essential to provide a flexible and adaptive interface that allows various parts to interact to achieve various advantages. An example would be the performance of consistent data exchange and information transfer between various systems. Because of the features provided by UAV mobility as they move in 3D space, the expanding range of services may necessitate the use of UAVs as gateways to numerous systems. It is critical to prepare the interacting components in such a complex system to ensure consistent interface quality.

### 7.5. Massive MIMO

Massive MIMO will revolutionize the way UAVs are used in communication networks. Massive MIMO guarantees several factors, such as the UAVs’ exceptional mobility. One scenario for massive MIMO in mobility is that a large number of antennas are used at the BS to serve multiple single–antenna terminals with very high capacity at the same time. As stated in [87], UAV deployment should not have major limits, which is what pilots prefer. Establishing enormous antennas for UAVs is a target that must be achieved to implement 5G connected UAVs in fully loaded networks without affecting performance for existing ground users. Several studies conducted on MIMO are cited here to provide researchers with the relevant knowledge [13,14,122,123,124,125,126,127,128].

### 7.6. Synergy of UAVs and IoT Systems

The Internet of UAVs (IoUAVs) is the dynamic integration of current IoT and UAVs. IoUAVs is a promising arrangement for creating the future IoT environment in which people, UAVs, and IoT gadgets are all harmoniously connected. This allows omnipresent data sharing and fine–granularity coordination among a swarm of UAVs due to unique features such as quick sending, simple programmability, controllable mobility, and flexibility. One of the technological contradictions is that while there are numerous benefits in IoUAVs application that arise from linking everything that can be connected, these operations necessitate significant energy capacity. UAVs are limited by their size, weight, and power (SWAP). SWAP limits have a direct impact on each UAV’s maximum operating altitude, communication, coverage, computation, and endurance capabilities, and IoUAVs are no exception. As a result, there is an urgent need to develop this aspect of IoUAVs to provide seamless mobility and connectivity. In [129,130,131], the authors employed a UAV that was dispatched to collect data from IoT devices under stringent time limitations. The total number of IoT devices was maximized by optimizing the UAV trajectory and wireless resource allocation simultaneously. They proposed a UAV trajectory planning algorithm that addresses mixed–integer nonconvex and difficult issues.

### 7.7. Full Duplex Communication

In [114,115,116,132], recent advancements in electronics, sensors, and communication systems have made the use of small UAVs possible for many various applications. However, small UAVs are insufficient. Multiple–UAVs can make create a system that is beyond the limitations of a single small UAV. FANETs can expand connectivity and communication range in infrastructure–less areas due to their mobility, lack of central control, self–organization, and ad–hoc nature. FANETs can provide a rapidly deployable, flexible, self–configurable, and relatively low–cost network in catastrophic situations; however, connecting multiple UAVs in ad–hoc networks is difficult. If some of the several UAVs are disconnected during the operation of a FANET due to weather conditions, they can still connect to the network via other UAVs. Furthermore, ad–hoc networking among UAVs, can solve complications such as short range, network failure, and limited guidance that arise in a single UAV system. Although such distinguishing characteristics make FANETs an appropriate solution for a variety of scenarios, they also introduce several challenging issues, such as communications and networking of multiple UAVs. This level of coordination requires a reliable communication architecture and routing protocols on highly dynamic flying nodes. Military applications, disaster response, and other uses for FANETs are some examples. Another potential application for UAVs is discussed; the Flying Ad–Hoc Network (FANET). UAV hubs are equipped with cameras and sensors that allow them to communicate and share data. Military applications, disaster response, and other uses for FANETs are just a few examples. The engineering of FANETs was further investigated to propose a new steering convention. A clustering calculation was also suggested to accelerate the execution of UAV systems.

### 7.8. Security and Privacy

The integrated network may be vulnerable to malicious attacks due to open connections and congested topologies that span a mission–critical range via purposeful jamming or disruptions. Since UAVs are constantly unattended, they can be easily seized or assaulted. Security is a critical issue in UAV–assisted systems. A secure and lightweight component is required to avoid malicious modification, such as eavesdropping, man–in–the–middle attacks, etc. To address cyber–physical security gaps in UAV communication systems, a zero–sum network interdiction game was created. The system considers the case of a vendor and an attacker trying to move UAVs from one point to another. This game can successfully ensure the cyber security of the UAV delivery system. Fake signal solutions were also suggested to keep UAVs safe in cellular–connected applications. A spoofer strategy can be used to create fake GPS signals that are almost indistinguishable from original GPS signals, making it more difficult for cyber attackers to hack into the system. Within the vast scope of space–air–ground coordinate systems, Software–defined networking (SDN) controllers are capable of overseeing assets and controlling operations. It is critical to protect SDN controllers from various cyber–attacks that allow adversaries to wiretap data and control signals transmitted through UAV framework radio connections. Cyber–attacks on UAV frameworks have been documented. Cyber–security is still a major issue in the real–world application of UAVs. Convenient tactics and counter–mechanisms must be planned ahead of time to counteract dangerous cyber–attacks. The important point to emphasize here is that the mobility of UAVs must be controlled by security. This keeps the routing positions under network management. Furthermore, the deployment points of the deployed UAV as UEs or BSs must be secure to prevent any attacks aimed at stealing users’ communication data.

## 8. Conclusions

Due to rapid technological advancements, UAVs have grown increasingly popular, attracting an increasing amount of attention in the field of wireless networks. Numerous articles on UAV–based network architectures have been included in the literature review. Particular attention was given to the development of HO for UAVs as well as the expansion of networks that make use of UAV technology. Several aspects of HO were considered by examining various available studies. Several key research problems, including 3D deployment and energy efficiency, were discussed. New methods to resolve the mentioned issues have been introduced, including algorithm–based learning, experimental works, etc. The challenges, potential solutions, and future research directions were examined. The fundamental problem with UAV–connected wireless networks is their 3D mobility. This study provides comprehensive information on the shift from the standard 2D mobility and 3D mobility to 5G and 6G networks. A conceptual explanation of numerous elements was also highlighted to aid in identifying the optimum HO decision.

## Figures and Tables

**Table 1:** HO procedure list.

HO Procedure	Description
Source—inter evolved Node BS (eNB) HO	This occurs when the user leaves the coverage area of eNB and enters another area covered by another eNB (within E–UTRAN).
User—inter eNB HO	This occurs when the user enters a coverage area managed by eNB to one that is managed by another eNB (within E–UTRAN).
Source—Inter RAT HO	This occurs when the user leaves the E–UTRN cell.
User—Inter RAT HO	This occurs when the user enters the E–UTRN cell.
Source—intra eNB HO	This occurs from one sector to another when the user leaves the sector.
User—intra eNB HO	This occurs from one sector to another when the user enters the sector.

**Table 2:** Summary of research challenges throughout the literature.

No	Challenge Group	Summary of Challenges
1	General and main challenges of connected UAVs	Connected UAV technology is used to place unmanned airships in situations where a human pilot cannot be placed due to risks. Maintenance personnel can employ UAS to conduct an initial inspection from the ground, avoiding perilous climbs and reducing casualties. The key concerns here are the risks connected with monitoring airborne applications. Pilot preparation, flight length, weather conditions, and risk constraints are all significant factors to consider.
2	UAV operations in LTE	LTE technology is well suited to serve air vehicles, particularly at low altitudes, and this provides great potential for the rapid growth in the number of UAVs in use. This, in turn, creates numerous commercial opportunities for modern communications, which consequently requires improvements to LTE networks in the future to readily serve the anticipated rapid growth of aircraft.
3	Mobility in 3D	Aerial and ground UEs are based on different assumptions. UAVs for network services are different from traditional networks in that they use a 3D model rather than a 2D model. UAVs are incredibly mobile, making control and decision–making difficult. As a result, advanced mobility solutions will be required.
4	UAV–ground channels	One of the most complex design difficulties in producing cellular–connected UAVs is creating coexisting mechanisms between terrestrial and airborne users. UAV–ground interference management must be installed to achieve this coexistence. The communication channel between the ground BS and UAVs has extremely distinct interruption patterns. The elevation or angle of the UAV influences channel parameters such as shadowing and path loss exponents. These can be used in residential or sub–residential environments, depending on deployment.
5	Transmission protocols	UAVs can scan and capture data while dropping data packets, according to several patent applications. Transmission Control Protocol/Internet Protocol (TCP/IP) will be insufficient for UAVs. As a result, new methods based on UAV mobility must be devised.
6	Dominance of LoS	When aerial and terrestrial users work together, UAVs cause considerable BS disturbance. Existing UAV HO experiments have shown to possess several shortcomings. Due to their high mobility, UAVs are frequently susceptible to HOs and the ping–pong effect.

## Abbreviations

2D	Two Dimensional
3D	Three Dimensional
4G	4th Generation
5G	5th Generation
6G	6th Generation
AP	Access Point
API	Application Programing Interface
BS	Base Station
BVLoS	Beyond Visual Line of Sight
D2D	Device–To–Device
DTN	Delay Tolerant Networking
FAA	Federal Aviation Administration
GPS	Global Positioning System
HetNets	Heterogeneous Networks
HO	Handover
H–RRM	HO and Radio Resource Management
ICIC	Inter–Cell Interference Coordination
LC	Loaded Cells
LoS	Line of Sight
UDCUN	Ultra–Dense Cloud–UAV Network
UDN	Ultra–Dense Networks
UE	User Equipment
UL	Uplink
UxNB	UAV–Mounted Bs
LTE	Long Term Evolution
LWA	Leaky–Wave Antenna
mcMTC	Mission–Critical Machine–Type Communication
MIMO	Multiple Input Multiple Output
mm–wave	Millimeter Waves
OTT	Over–The–Top
Pf	False Ho Initiation Probability
Ps	Seamless Ho Success Probability
QoS	Quality of Service
RLF	Radio Link Failure
eNB	evolved Node BS
UAV	Unmanned Aerial Vehicle
RSS	Received Signal Strength
SDN	Software–Defined Networking
SINR	Signal–To–Interference–Plus–Noise Ratio
SWAP	Size, Weight, And Power
THz	Terahertz
TCP/IP	Transmission Control Protocol/Internet Protocol
FANET	Flying Ad–Hoc Network
VR	Virtual Reality
WLAN	Wireless Local Area Network
RWP	Random Waypoint
WWAN	Wireless Wide Area Network
RW	Random Walk
